# Mutation Profiles of eGFP-Tagged Small Ruminant Morbillivirus During 45 Serial Passages in Ribavirin-Treated Cells

**DOI:** 10.3389/fvets.2021.690204

**Published:** 2021-07-21

**Authors:** Fuxiao Liu, Yanli Zou, Lin Li, Chunju Liu, Xiaodong Wu

**Affiliations:** ^1^College of Veterinary Medicine, Qingdao Agricultural University, Qingdao, China; ^2^OIE Reference Laboratory for Peste des Petits Ruminants, China Animal Health and Epidemiology Center, Qingdao, China

**Keywords:** rSRMV-eGFP, next-generation sequencing, single-nucleotide variation, mutation, L protein, high-fidelity

## Abstract

Small ruminant morbillivirus (SRMV), formerly known as peste-des-petits-ruminants virus, classified into the genus *Morbillivirus* in the family *Paramyxoviridae*. Its L protein functions as the RNA-dependent RNA polymerases (RdRp) during viral replication. Due to the absence of efficient proofreading activity in their RdRps, various RNA viruses reveal high mutation frequencies, making them evolve rapidly during serial passages in cells, especially treated with a certain mutagen, like ribavirin. We have previously rescued a recombinant enhanced green fluorescence protein-tagged SRMV (rSRMV-eGFP) using reverse genetics. In this study, the rSRMV-eGFP was subjected to serial passages in ribavirin-treated cells. Due to the ribavirin-exerted selective pressure, it was speculated that viral progenies would form quasispecies after dozens of passages. Viral progenies at passage-10, -20, -30, -40, and -50 were separately subjected to next-generation sequencing (NGS), consequently revealing a total of 34 single-nucleotide variations, including five synonymous, 21 missense, and one non-sense mutations. The L sequence was found to harbor eight missense mutations during serial passaging. It was speculated that at least one high-fidelity variant was present in viral quasispecies at passage-50. If purified from the population of viral progenies, this putative variant would contribute to clarifying a molecular mechanism in viral high-fidelity replication *in vitro*.

## Introduction

Peste des petits ruminants is a highly contagious disease, mainly affecting goats and sheep, and even large ruminants (1–3). Its etiological agent is small ruminant morbillivirus (SRMV), formerly known as peste-des-petits-ruminants virus. SRMV belongs to the genus *Morbillivirus* in the family *Paramyxoviridae*. Its genome is a single strand of RNA with negative polarity (4), coding for six structural proteins, namely nucleocapsid (N), phospho (P), matrix (M), fusion (F), hemagglutinin (H), and large (L) proteins in the order of 3′-N-P (V/C)-M-F-H-L-5′. The V and C proteins are two non-structural proteins, encoded through alternative expression strategies from the P gene transcription unit (5). Viral RNA genome is encapsulated by the N protein forming a helical nucleocapsid, in combination with the L and P proteins to form a ribonucleoprotein complex (6). The L protein is an RNA-dependent RNA polymerase (RdRp), the largest of the morbilliviral proteins, and is least abundant in virus-infected cells. It is assumed to carry all activities necessary for genomic RNA transcription and replication (5).

The overwhelming majority of RNA viruses use their own low-fidelity RdRps to initiate RNA synthesis (7–9), consequently generating viral quasispecies consisting of a swarm of viruses. Compared with most DNA viruses, morbilliviruses are characterized by high mutation frequencies in their genomes and antigenomes during viral serial passages *in vitro*, mainly attributed to the lack of effective proofreading activities in their RdRps (10). For example, the measles morbillivirus was estimated to have a mutation rate of 9 × 10^−5^/nt/replication and a genomic mutation rate of 1.43/replication by analysis on the frequency of monoclonal antibody-resistant mutants (11). More recently, Zhang et al. (12) determined a spontaneous mutation rate of 1.8 × 10^−6^/nt/replication in measles morbillivirus under non-selective conditions.

Using Sanger sequencing, we have reported major mutation events in structural genes of a China SRMV isolate (China/Tibet/Geg/07-30, GenBank: FJ905304.1) during 90 serial passages *in vitro* (13). However, the conventional Sanger sequencing is not capable of generating a large dataset to identify all single-nucleotide variations (SNVs) in viral progenies. As an alternative technique, the next-generation sequencing (NGS) has been broadly applied to analyze and to quantify the exceptionally-high diversity within viral quasispecies. By means of it, single-nucleotide polymorphisms (SNPs) in a variety of viruses can be systematically analyzed to reveal their evolutionary profiles (14–18).

Enhanced green fluorescence protein (eGFP) is an ideal marker, which facilitates screening of high-fidelity mutants to unveil a given mechanism concerning high-fidelity replication of RNA virus (19). We have recovered an eGFP-tagged recombinant SRMV (rSRMV-eGFP) from the cDNA clone of Nigeria 75/1 vaccine strain (20). In the present study, the rSRMV-eGFP was subjected to 45 serial passages in Vero-Dog-SLAM (VDS) cells. In order to quicken the process of SNVs in the rSRMV-eGFP genome, VDS cells were always treated with ribavirin, a widely used mutagen that could force RNA viruses into mutagenesis, and even into error catastrophe (21, 22). The NGS technique was used to uncover the mutation profiles of viral progenies at different passages.

## Materials and Methods

### Cell and Virus

The VDS cell line was cultured at 37°C with 5% CO_2_ in Dulbecco's modified Eagle's medium (DMEM) supplemented with 10% fetal bovine serum (FBS), and containing penicillin (100 U/mL), streptomycin (100 μg/mL), amphotericin B (0.25 μg/mL), and G418 (500 μg/mL). The rSRMV-eGFP was rescued previously in our laboratory (20).

### Forty-Five Serial Passages of rSRMV-eGFP

The passage-5 (P5) rSRMV-eGFP was serially passaged (3 d/passage) in ribavirin (Solarbio, Beijing, China)-treated VDS cells at undetermined MOI values for 45 passages (P6 to P50) in the biosafety level-3 laboratory of China Animal Health and Epidemiology Center. The ribavirin concentration was gradually increased in DMEM with passaging: 80 μM during P6–16, 160 μM during P17–24, 240 μM during P25–32, 320 μM during P33–42, and 400 μM during P43–50. Virus-infected cells were observed under a fluorescence microscope before each round of passaging.

### NGS Analyses of P10, P20, P30, P40, and P50 Progenies

Culture supernatants with rSRMV-eGFP-infected cells were independently harvested from the P10, P20, P30, P40, and P50 progenies for extracting viral RNA using the Viral RNA/DNA Extraction Kit (Takara, Dalian, China), according to the manufacturer's instruction. A total of five RNA samples were separately reverse transcribed using the 1st Strand cDNA Synthesis Kit (Takara, Dalian, China) and random hexamers, according to the manufacturer's instruction. The Illumina sequencing and library construction were performed as descried previously (18). In brief, the NEBNext® Ultra™ II RNA Library Prep Kit (NEB, Ipswich, MA, USA) was used for library construction. After adapter ligation, 10 cycles of PCR amplification were performed for sequencing target enrichment. The libraries were pooled at equal molar ratio, denatured, and diluted to optimal concentration prior to sequencing. The Illumina NovaSeq 6000 (Illumina, San Diego, CA, USA) was used for sequencing to generate pair-end 150 bp reads.

### Processing and Analysis of NGS Data

Processing and analysis of NGS data were also carried out as descried previously (18). In brief, raw reads were filtered by fastp (https://github.com/OpenGene/fastp) to remove sequencing adapters and low-quality reads, including those reads scored < Q20. Ribosomal RNAs and host reads subtraction by read-mapping were performed with BBMap program (https://github.com/BioInfoTools/BBMap). A *de-novo* genome assembly was performed using SPAdes v3.14.1 (https://github.com/ablab/spades). These extracted assembled scaffolds limited the minimum contig length to 100 bases, with the best BLAST hits to the NCBI nucleotide database. High-quality filtered reads were mapped against the rSRMV-eGFP reference genome by Burrows-Wheeler Aligner v0.7.17 (http://bio-bwa.sourceforge.net/), which also generated a BAM file to calculate the mapping depth and coverage. SNPs were identified using an integrated software package, Snippy v4.4.5 (https://github.com/tseemann/snippy), which included both substitutions and insertions/deletions. The available SNP results were selected if mapping quality was ≥ 60 and depth was ≥ 10.

## Results

### Fluorescence-Attenuated or -Disappeared Syncytia Become Visible With Passaging

**Figure 1:**
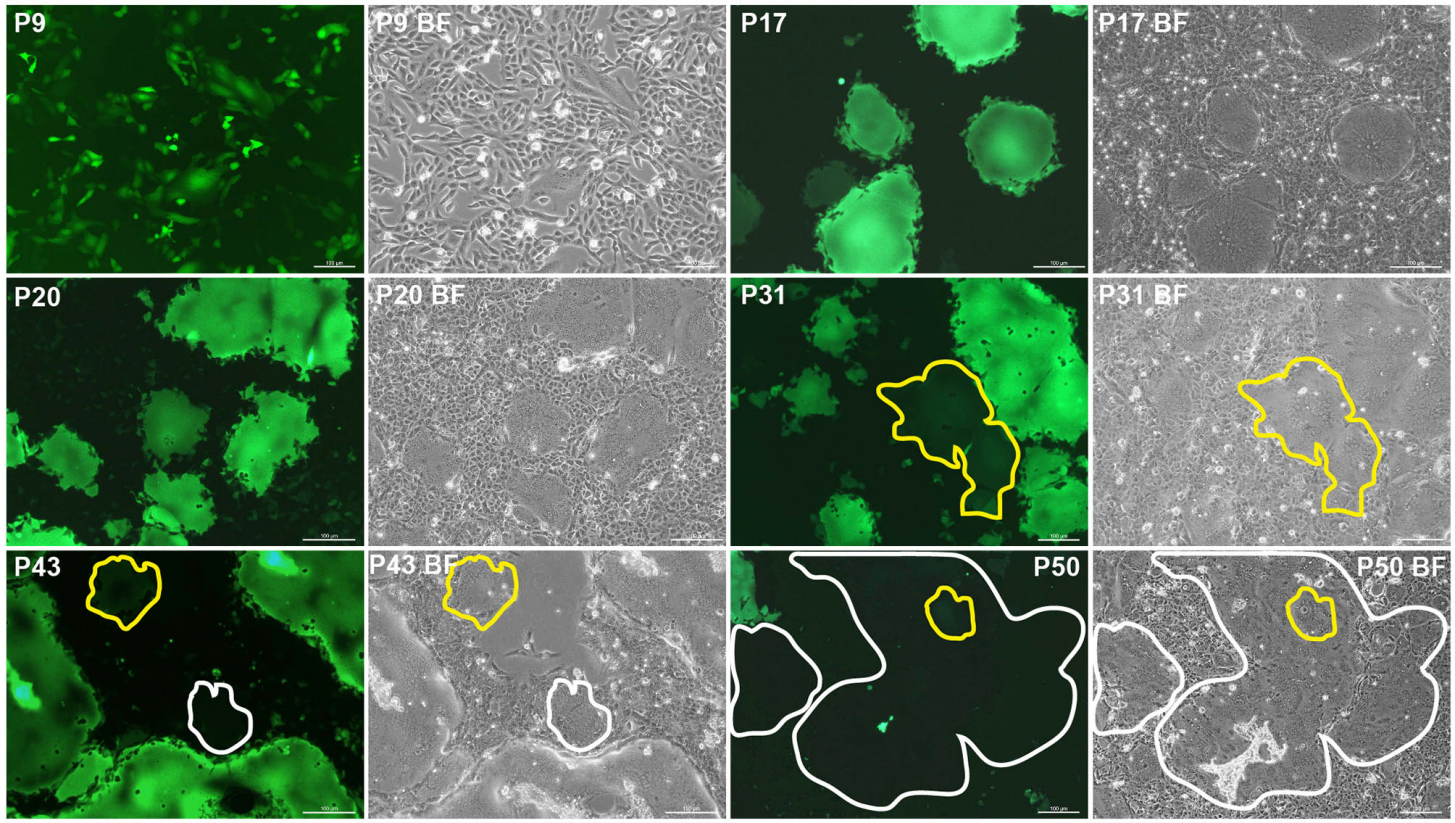
Fluorescence-attenuated or -disappeared syncytia formation during 45 serial passages in ribavirin-treated VDS cells. Fluorescence-attenuated and -disappeared syncytia are enclosed by yellow and white lines, respectively. BF, bright field.

The P5 rSRMV-eGFP was serially passaged in ribavirin-treated VDS cells. Fluorescent syncytia were always observable during passaging (Figure 1). However, a few fluorescence-attenuated syncytia occasionally appeared on cell monolayers approximately after the 24th passaging (Figure 1, enclosed by yellow line at P31), implying that the eGFP open reading frame (ORF) was undergoing missense mutations, or nucleotide deletions from the viral genome. A few fluorescence-disappeared syncytia (Figure 1, enclosed by white line at P43) became visible at P43 but were uneasily found on the virus-inoculated cell monolayer at 72 h post-infection (hpi). The proportion of fluorescence-disappeared syncytia increased with passaging and even was predominant in a randomly selected field-of-view of fluorescence microscope at P50 (Figure 1, enclosed by white line).

### NGS Shows Analyzable Sequencing Depths

**Figure 2:**
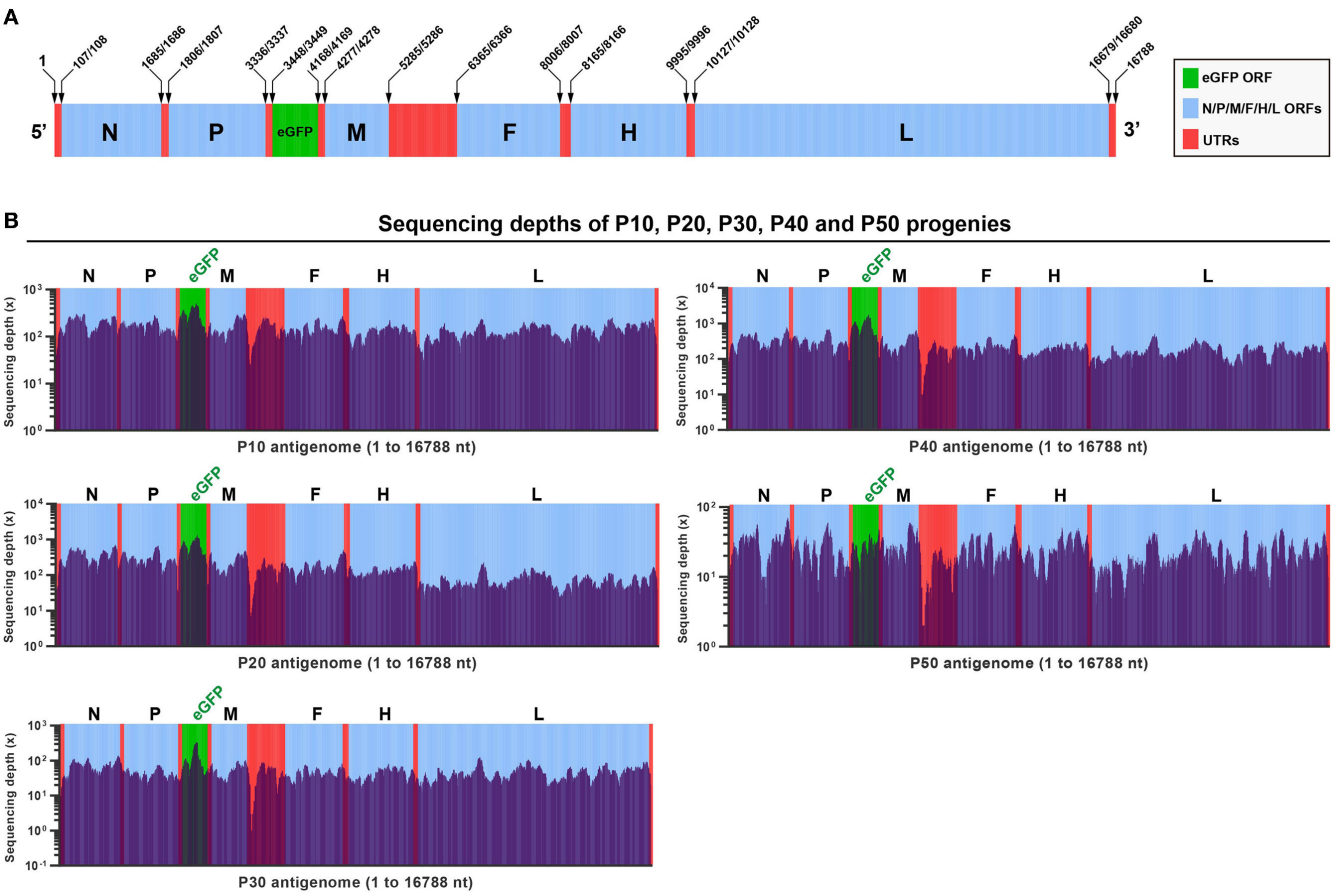
Analyses on next-generation sequencing of rSRMV-eGFP progenies. Schematic representation of rSRMV-eGFP antigenome **(A)**. All elements proportionally match their actual lengths in viral antigenome. ORF: open reading frame; UTR: untranslated region. Depth and coverage of next-generation sequencing across the rSRMV-eGFP antigenome at P10, P20, P30, P40, and P50 **(B)**. All elements proportionally match their actual lengths in viral antigenome.

The rSRMV-eGFP had a 16788-nt-long genome. Figure 2A schematically showed all ORFs and untranslated regions (UTRs) in proportion to their actual distributions in the viral antigenome. To uncover viral mutation profiles with passaging, a total of five RNA samples were independently extracted from the P10, P20, P30, P40, and P50 culture supernatants, and then subjected to the NGS analysis. The NGS data were deeply analyzed to yield acceptable sequencing depths for the P10, P20, P30, and P40 samples, but the P50 sample that showed the average sequencing depth at a low level (Figure 2B). All five samples were determined to have high (>99.8%) coverage ranges across the full-length antigenome sequence. Uncovered regions were located at 5′- and 3′-end regions of the antigenome. The eGFP was measured with relatively high sequencing depths at the P10, P20, P30, and P40, whereas a short GC-rich region between the M and F ORFs showed the lowest depths for all five samples (Figure 2B).

### Thirty-Four SNVs Are Identified in Five Progenies

**Figure 3:**
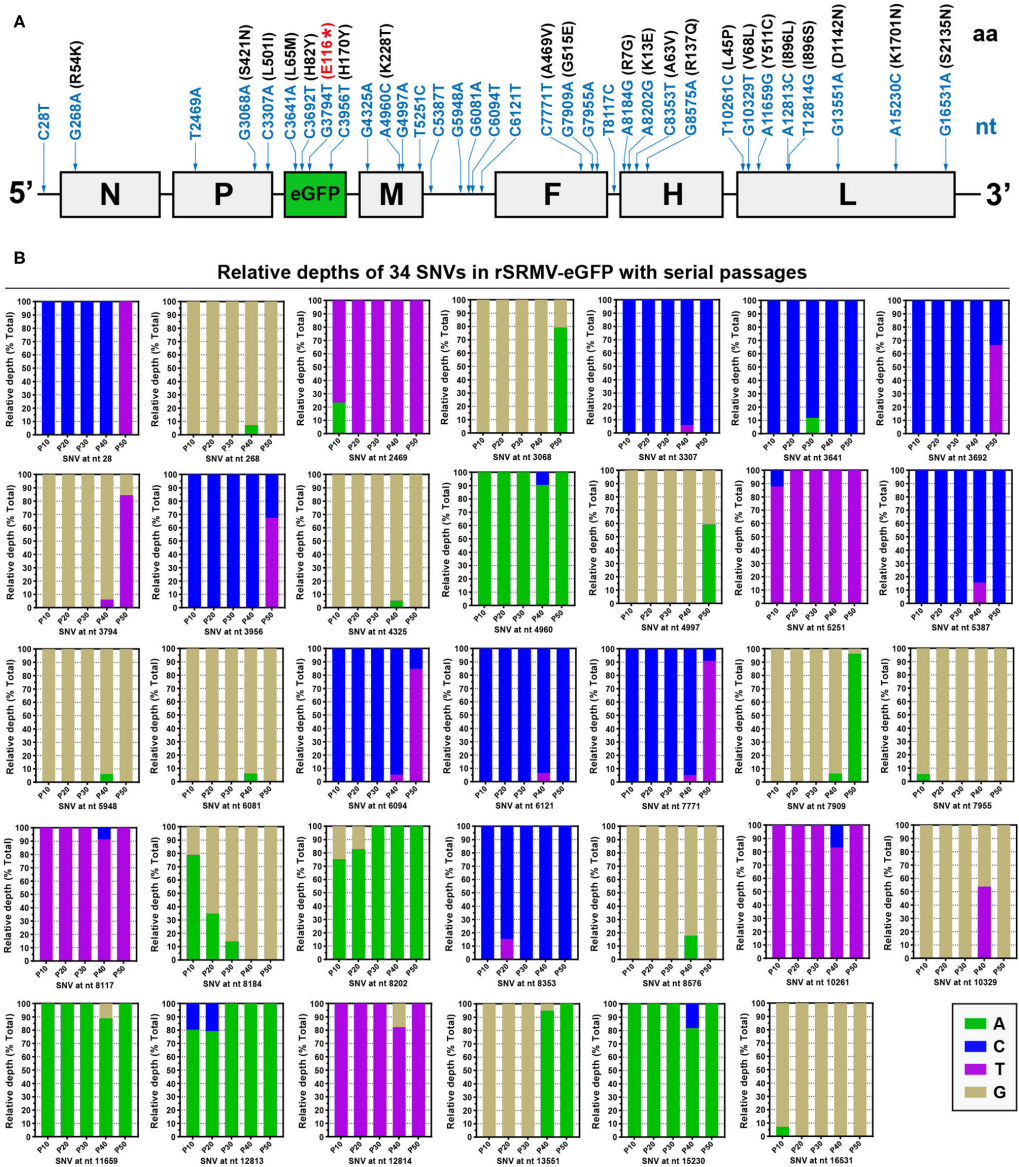
Progeny profiles of nucleotide and amino acid mutations at P10, P20, P30, P40, and P50. All detectable single-nucleotide and -amino acid mutations in rSRMV-eGFP antigenome during passaging **(A)**. The positions of SNVs are independently indicated with blue arrows. SNVs are marked with blue letters and numbers. Missense mutation-caused amino acid substitutions are marked with black letters and numbers in black brackets. The non-sense mutation, G3794T, causes early termination of eGFP translation, marked with “(E116*)” in red. All elements do not match their actual lengths in proportion. Relative depths of next-generation sequencing for 34 SNVs in rSRMV-eGFP antigenome with 45 serial passages **(B)**. The relative depth is referred to as the proportion of sequencing depth of a given nucleotide to the total.

The NGS analyses showed that neither sequence-deleting nor -inserting phenotype was detectable in the five progenies, within which a total of 34 SNVs arose with passaging, containing 26 transitions and eight transversions. The positions of SNVs were independently indicated with blue arrows in the rSRMV-eGFP antigenome, in which neither ORFs nor UTRs proportionally matched their actual lengths (Figure 3A). Figure 3B revealed complete profiles of 34 SNVs during viral serial passages. Each SNV was identified as a SNP with mixture of two nucleotides. The 5′ UTR exhibited one SNV (C28T) only at P50. The other two UTRs, between the M and F ORFs as well as between the F and H ORFs, exhibited five and one SNVs, respectively. Except the C28T and C6094T at P50, the other SNVs showed low frequencies of occurrence in UTRs. Seven ORFs, namely the N, P, eGFP, M, F, H, and L, showed one, three, four, four, three, four, and eight SNVs, respectively. Several SNVs, such as T2469A and T5251C, appeared at a given passage, but reverted back to their original statuses with passaging. In contrast, the site at nt 8184 underwent gradual substitution of a nucleotide for another one with passaging. Its original nucleotide was “A,” which was gradually and finally replaced by “G” at P40. There were some low-frequency point mutations, like G5948A and G6081A, occurring once only at a single passage.

### Synonymous, Missense, and Non-Sense Mutations Are Identified in Antigenome

Seven ORFs totally showed 27 of their own SNVs, including five synonymous, 21 missense, and one non-sense mutations. Accordingly, 21 missense mutations caused 21 amino acid (aa) mutations (Figure 3A, black-marked in black brackets), distributed in seven protein sequences. The L protein showed the most aa mutations, up to eight. In the L protein sequence, the SNV “A12813C” occurred at P10 and P20, and its adjacent SNV, “T12814G,” arose at P40. The “A12813C” and “T12814G” led to substitutions of leucine and serine for the same isoleucine at aa 896, respectively. As a non-self-sequence, the eGFP ORF harbored a non-sense mutation at nt 3794, causing the early termination of translation at aa 116 in the eGFP sequence (Figure 3A, red-marked in red bracket). The SNV “G3794T” was initially identified at P40, when it only showed a low frequency of occurrence. Nevertheless, this SNV became predominant in viral quasispecies at P50 (Figure 3B), directly responsible for a number of fluorescence-disappeared syncytia that appeared on the P50 cell monolayer at 72 hpi.

## Discussion

The SRMV Nigeria 75/1 strain has been widely used for vaccine production. Its reverse genetics system had been constructed previously in our laboratory for further use to rescue recombinant SRMVs expressing foreign proteins (20, 23, 24). The rSRMV-eGFP was demonstrated to have a similar growth curve to that of the wide-type strain (20), implying no significant interference of the eGFP with viral replication. Moreover, the eGFP facilitated observation of fluorescent syncytium formation on a cell monolayer in real time. Such a feature prompted us to use the rSRMV-eGFP for uncovering viral mutation profiles during serial dozens of passages. To promote occurrence of SNVs in the eGFP ORF, VDS cells were treated with the ribavirin, which could act as a viral mutagen forcing RNA viruses into mutagenesis. In order to make the rSRMV-eGFP gradually adapt to the ribavirin-treated cells, the ribavirin concentration was progressively increased with viral passaging. The rSRMV-eGFP was subjected to 45 serial passages in ribavirin-treated cells and speculated to form a rich diversity of viral quasispecies at P50, because most RNA viruses are genetically unstable during replication (8).

To explore SNV profiles of the rSRMV-eGFP during serial passages, the P10, P20, P30, P40, and P50 progenies were collected for the NGS analysis. The major reason why the conventional Sanger sequencing was not used in this study was its limited ability or inability to identify low-frequency mutations, seriously reducing its applicability to quantify the complexity of mutant spectra. In contrast, the NGS, because capable of generating a large dataset for identification of SNMs in viral genomes, has been widely used to analyze quantitatively the rich diversity of viral quasispecies (18, 25, 26).

Viral self-proteins are intrinsically encoded in rSRMV-eGFP-infected cells. In order to avoid the impact of an error catastrophe on virus propagation, harmful or especially lethal SNVs would not accumulate in viral self-sequences. Indeed, the NGS analyses revealed no lethal SNV in the viral self-sequences. In contrast, the SNVs were unrestricted in the eGFP ORF during viral propagation, due to the eGFP as a foreign protein, theoretically uninvolved in a series of SRMV-related events, for example, replication, transcription, regulation, and packaging. In other words, SNVs would be random, uncontrolled, and retainable in the eGFP ORF during viral propagation. Indeed, compared with the other ORFs in the rSRMV-eGFP antigenome, the eGFP sequence revealed a high mutation frequency, as evidenced by the NGS analyses.

A total of four SNVs, including one non-sense mutation, appeared in the eGFP ORF during serial passages. The first one (C3641A) was found to arise at P30, causing substitution of methionine for leucine at aa 65. It remained unclear whether the “L65M” resulted in the fluorescence-attenuated syncytium formation on cell monolayer at P31 (Figure 1, P31). The unique non-sense mutation arose in the eGFP ORF at P40, directly causing the early termination of eGFP expression. Consequently, fluorescence-disappeared syncytia were always observable after P40, and even predominant on cell monolayer at P50. In addition, the NGS analysis revealed the non-sense mutation with a high frequency of occurrence at P50. Even so, fluorescent syncytia were still visible at P50 (Figure 1, P50) and possibly induced by a high-fidelity mutant. If such a mutant exists in viral quasispecies at P50, its high-fidelity mechanism would be attributed to the mutated L protein, because RdRp is a primary driving force of point mutations observed in RNA virus populations (7).

Historically, a virulence-attenuated canine morbillivirus (Rockborn strain) was reported to revert back to virulent form after serial passages in dogs (27). Although there was no similar report about reversion to SRMV virulence *in vivo*, Abdullah et al. (28) recently demonstrated that change of a single aa was sufficient for causing SRMV to enter human cells. Therefore, screening of a high-fidelity SRMV vaccine strain would play an important role not only in immunization of animals, but also in reduction of a potential risk in viral trans-species transmission. In this study, the L sequence was found to harbor eight missense mutations during serial passaging, out of which six were identified at P40. Unexpectedly, the P50 progeny showed no SNV in the L ORF, mainly attributed to too low sequencing depths to exhibit some low-frequency mutations. We speculated that at least one high-fidelity mutant was present in viral quasispecies at P50. If purified from the population of viral progenies, this putative mutant would contribute to clarifying a molecular mechanism in viral high-fidelity replication *in vitro*.

## Data Availability Statement

The data presented in the study are deposited in the NCBI SRA repository, accession number (PRJNA736161).

## Author Contributions

FL: conceptualization and writing—original draft preparation. YZ: methodology. LL and CL: formal analysis. XW: project administration. All authors contributed to the article and approved the submitted version.

## Conflict of Interest

The authors declare that the research was conducted in the absence of any commercial or financial relationships that could be construed as a potential conflict of interest.
